# Access to school-based eye health programs: a qualitative case study, Bogotá, Colombia

**DOI:** 10.26633/RPSP.2021.154

**Published:** 2021-12-16

**Authors:** Aryati Yashadhana, Nina Serova, Ling Lee, Luisa Casas Luque, Leonardo Ramirez, Juan Carlos Silva, Anthea M Burnett

**Affiliations:** 1 Brien Holden Vision Institute Brien Holden Vision Institute Sydney Australia; 2 Pan American Health Organization Pan American Health Organization Bogota Colombia

**Keywords:** Eye health, vision screening, schools, equity, Colombia, Salud ocular, selección visual, instituciones académicas, equidad, Colombia, Saúde ocular, seleção visual, instituições acadêmicas, equidade, Colômbia

## Abstract

**Objectives.:**

To identify barriers and enablers to accessing school-based eye health programs in Bogotá, Colombia.

**Methods.:**

We undertook a qualitative case study that explored how structural factors, and social and cultural norms influence access to school-based eye health programs. We conducted focus groups discussions and interviews with a purposive sample of 37 participants: government stakeholders (n = 4), representatives from nongovernmental organizations (n = 3), and an eye-care practitioner, as well as teachers (n = 7), a school nurse, parents (n = 7), and children (n = 14) from private and public schools. Data were analyzed using a priori themes from the availability, accessibility, acceptability and quality framework.

**Results.:**

Routine vision screening in schools is not currently provided nor is there a budget to support it. Lack of collaboration between the health and education ministries and the absence of national planning affected the delivery of eye care in schools. Factors related to acceptability of school-based eye health programs included: poor acceptance of training teachers as vision screeners; stigma related to wearing spectacles; and distrust of health services. The cost of spectacles and poor access to eye health information were identified as barriers to positive child eye health outcomes by socioeconomically disadvantaged parents and children.

**Conclusion.:**

Our findings suggest the need for a national school eye health plan and improved cooperation between health and education ministries. Interventions to improve trust in health services, tackle the lack of human resources while respecting professional qualifications, and raise awareness of the importance of eye health are recommended.

## RESUMEN

**Objetivo.:**

Determinar los obstáculos y los elementos facilitadores para acceder a los programas de salud ocular en las escuelas en Bogotá (Colombia).

**Métodos.:**

Se llevó a cabo un estudio cualitativo de casos que exploró cómo influyen los factores estructurales y las normas sociales y culturales en el acceso a la atención oftalmológica. Se realizaron entrevistas y grupos de opinión con una muestra intencionada de 37 participantes: interesados directos gubernamentales (n = 4), representantes de organizaciones no gubernamentales (n = 3) y un profesional de atención oftalmológica, así como docentes (n = 7), una enfermera escolar, padres (n = 7) y niños (n = 14) de escuelas privadas y públicas. Se analizaron los datos empleando temas determinados a priori sobre la disponibilidad, la accesibilidad, la aceptabilidad y el marco de calidad.

**Resultados.:**

Actualmente, no se proporciona un tamizaje sistemático de la visión en las escuelas, ni se dispone de presupuesto para respaldarlo. La falta de colaboración entre los ministerios de salud y de educación, y la falta de planificación a nivel nacional tuvo repercusiones sobre la prestación de atención oftalmológica en las escuelas. Entre los factores relacionados con la aceptabilidad de los programas de salud ocular en las escuelas se encuentran una escasa aceptación de la posibilidad de capacitar a los docentes para que examinen la visión; el estigma relacionado con el uso de anteojos y la desconfianza de los servicios de salud. Los padres y los niños en una situación socioeconómica desfavorable indicaron que el costo de las gafas y el acceso limitado a la información sobre la salud ocular eran obstáculos para obtener resultados positivos de salud ocular en los niños.

**Conclusiones.:**

Los resultados indican la necesidad de un plan nacional de salud ocular en las escuelas y una mejor cooperación entre los ministerios de salud y de educación. Se recomiendan intervenciones para mejorar la confianza en los servicios de salud, abordar la falta de recursos humanos al tiempo que se respetan las cualificaciones profesionales y concientizar sobre la importancia de la salud ocular.

## RESUMO

**Objetivos.:**

Identificar barreiras e facilitadores para o acesso a programas escolares de saúde ocular em Bogotá, Colômbia.

**Métodos.:**

Estudo de caso qualitativo que explorou como fatores estruturais e normas socioculturais influenciam o acesso aos cuidados com a visão. Realizamos discussões em grupos focais e entrevistas com uma amostra intencional de 37 participantes: partes interessadas do governo (n = 4), representantes de organizações não governamentais (n = 3) e um oftalmologista, bem como professores (n = 7), uma enfermeira escolar, pais de alunos (n = 7) e alunos (n = 14) de escolas públicas e particulares. Os dados foram analisados de acordo com temas escolhidos a priori, a partir do marco de disponibilidade, acessibilidade, aceitabilidade e qualidade.

**Resultados.:**

O exame de visão de rotina não é realizado atualmente nas escolas, nem há espaço orçamentário para fazê-lo. A falta de colaboração entre os Ministérios da Saúde e da Educação e a ausência de planejamento em escala nacional afetaram a prestação de cuidados oftalmológicos nas escolas. Fatores relacionados à aceitabilidade dos programas escolares de saúde ocular incluíram baixa aceitação do treinamento de professores para realizar triagem de problemas visuais, estigma relacionado ao uso de óculos e desconfiança nos serviços de saúde. O custo dos óculos e o acesso limitado a informações sobre saúde ocular foram identificados como barreiras para desfechos positivos de saúde ocular infantil em pais e crianças em situação de desvantagem socioeconômica.

**Conclusão.:**

Os resultados sugerem a necessidade de um plano nacional de saúde ocular nas escolas e de uma melhor cooperação entre os Ministérios da Saúde e da Educação. Recomendam-se intervenções para aumentar a confiança nos serviços de saúde, combater a falta de recursos humanos (respeitando as qualificações profissionais) e aumentar a conscientização sobre a importância da saúde ocular.

Uncorrected refractive error is one of the leading causes of vision impairment in children (1), which has been associated with poorer educational attainment (2, 3). Epidemiological research on the burden of refractive error in Latin America, and Colombia specifically, is limited. A school-based survey in Brazil found the prevalence of unaided vision impairment to be 4.8%, with 76.8% due to refractive error (4). Vision impairment has been recognized in Colombia as the second-highest cause of disability, with young people (aged 12–29 years) having the least access to appropriate support (5).

The International Covenant on Economic, Social and Cultural Rights (6), and the Convention on the Rights of the Child (7) of which Colombia is a party, define access to education and health care as a child’s human right. Importantly, equitable access to appropriate eye care for children is fundamental for achieving good quality, inclusive education, which is a key component of achieving Sustainable Development Goals (SDGs) 4 (quality education) and 10 (reduced inequalities) (8). The 2021 Lancet Global Commission on Global Eye Health (9) highlighted eye health as essential to achieving the SDGs, and to do so, vision needs to be reframed as a development issue. Furthermore, the Pan American Health Organization’s 2020 strategic plan specified the need to: reduce blindness and visual impairment in children (section 16d); increase available, accessible, attainable, and quality eye health services (section 16b); and ensure access to vision correction to enable their right to education (10). School-based eye care has the potential to provide high-quality and cost-effective services that enable early detection of eye diseases, vision impairment and blindness (11), thus preventing associated learning difficulties (12). School attendance in Colombia has been increasing (13), presenting a unique opportunity to detect vision impairment in children. However, peer-reviewed research on how eye-care services might be appropriately placed in Colombian schools is limited (11).

Colombia is classified as a middle-income country, and has a population of over 50 million people (14). Income inequality has led to a rise in urban slums and informal settlements, where residents experience barriers to health, education, and housing (15). Almost one sixth of the population lives in the capital city of Bogotá, which is classified into six social classes (strata), defined by socioeconomic variables (stratum one comprises people on the lowest incomes) (16). Strata classification determines the amount of government subsidy residents receive for health care (16), including optometry and ophthalmology consultations (17). Lower strata (one to two) have access to fully subsidized health care, while higher strata (three to six) are subject to co-payments. The Colombian Ministry of Health is responsible for overseeing child eye health, and its 10-year national plan aims to increase the management of vision impairment in early childhood, promote visual health nationally, identify refractive error in children between 2 and 8 years old, and treat all children identified (5). To reach this goal, the “I see well, I learn well” program, to be implemented by the local health programs, was launched in 2016 (17).

The purpose of this study was to explore school-based eye health programs in Colombian schools and identify key factors that inhibit or facilitate children’s access to school-based eye health programs. To explore the different dimensions of accessibility, we used the availability, accessibility, acceptability, quality (AAAQ) framework, which applies a rights-based approach to health system coverage, accessibility, and social determinants (18).

## METHODS

This study was conducted between 2017 and 2018, and aimed to identify barriers and enablers to accessing and benefitting from school-based eye health programs in Bogotá, Colombia. We used qualitative case study methodology (19) to explore how structural factors, as well as social and cultural norms, may influence access to school-based eye health programs. Our study did not aim to be representative, but rather to reflect the range of community characteristics within the identified research context.

### Participants

We used purposive stratified sampling methods (20) to recruit three groups of participants: community members (children and parents); school staff (teachers and principals); and key informants (representatives from government or nongovernmental organizations, and eye-care practitioners). We considered purposive sampling appropriate, as participants were recruited through existing relationships with in-country personnel. Two private schools (strata four and five) and one public school (strata two and three) participated in the study. To capture a range of perspectives and experiences, we asked school administrative staff to recruit children (aged 5–15 years) with spectacles (*n* = 4) and without (*n* = 10), and their parents, to the study.

Focus groups explored experiences and perspectives of school-based eye assessments, and, where relevant, children’s experience of wearing spectacles. We also conducted in-depth interviews with school staff (teachers, principals, and school nurses) and key informants, including government representatives from the Colombian Ministry of Health or Ministry of Education. Interviews explored policies, systems, and programs related to school-based eye care, including factors that enabled or obstructed access. The focus groups and in-depth interviews were conducted in Spanish, unless the participant(s) felt comfortable speaking in English. All interviews and focus groups were audio-recorded, transcribed verbatim and, where necessary, translated into English.

### Ethical considerations

Ethical clearance for the study was obtained from the research ethics committee at Antonio Nariño University, Bogotá. All participants were provided with an information statement (in Spanish) about the study and also a verbal explanation (in Spanish). We obtained written consent from all respondents before participation.

### Data analysis

The data were de-identified, transcribed, and analyzed using NVivo 11 software, version 11 (QSR International Pty Ltd, Australia). Thematic deductive coding (21) was undertaken to identify a priori themes from the AAAQ framework. The themes were also coded as either a barrier or facilitator to delivering school-based eye health programs where relevant. Any disagreements in coding were discussed and reviewed.

## RESULTS

The characteristics of the participants are shown in Table 1. Two child focus groups (public school *n* = 7, private school *n* = 7), one parent focus group (*n* = 6), and one parent interview were conducted. Child focus groups included children who wore spectacles (*n* = 4), children who had been prescribed with spectacles but were not wearing them (*n* = 3), and children who had not been prescribed spectacles (*n* = 8). We also conducted in-depth interviews with school staff (*n* = 7), including two principals, four teachers and one special education teacher, and key informants (*n* = 8) from government and non-government sectors. Selected quotations from participant interviews and focus groups that illustrate the themes described below are given in Table 2.

### Availability

**National health policies and budget.** Key informants identified key barriers to the delivery of school-based eye care at the policy level. In Colombia, no explicit school health policy exists, and routine vision screening in schools is not provided, nor is there a budget to support it. Government representatives noted that since 2015, the Ministry of Health has been working to create comprehensive service pathways and evidence-based interventions to detect, treat, and rehabilitate vision conditions. However, policies and budgets on school health were a disputed topic between the health and education ministries.

**TABLE 1.** Number of participants in the study of school-based eye programs, Bogotá, Colombia

Type of participant	Private school	Public school	Total
Schoolchildren (5–15 years)			
Male	0	3	3
Female	7	4	11
Parents			
Male	0	1	1
Female	6	0	6
School staff			
Principal	1	1	2
Teacher	2	3	5
School nurse	1	0	1
Key informants			
Government representative	NA	NA	4
Nongovernmental organization representative	NA	NA	3
Eye-care practitioner	NA	NA	1
**Total**	10	12	37

NA, not applicable.

***Source***: Prepared by the authors based on the analysis of the study data.

**TABLE 2.** Themes related to school-based eye programs and quotations from study participants, Bogotá, Columbia

AAAQ theme Subtheme	Selected quotations
**Availability**	
National health policies and budget	The Ministry of Health and the Ministry of Education, they are castles, the Ministry of Education don’t want the Ministry of Health coming in to do things. (NGO representative) The education sector has more resources assigned from the national budget than the health sector, almost 50% more resources. (Government representative) A lot of lobbying [is required] though. Convincing the EPS is not very useful because they are convinced of saving money to gain more. (NGO representative) We haven’t been able to establish the relationships between the ministries which could also mean there is no political will between them. Existing cooperation has been achieved through informal relationships. (Government representative)
Systems and resources to support school-based eye care	The school gives the list to secretary of health, secretary of health meets with EPS [who] contacts the parent directly. It’s happening but [the system] still needs more regulation. Not all schools embrace this policy. (Government representative) Teachers would do the screening and make a list that was then handed over to the health sector, and then the health sector did not follow through. (Government representative) We should do screenings, or even have an optometrist who comes to the school and teaches us how to detect signs and symptoms, but I think it’s mainly because of cost. (School nurse, private school) It is better when non-government institutes provide eye health services. This is a public school, most of the parents don’t have the economic capacity. The results aren’t the same through the health system. Thanks to non-government organizations the barriers are fading a little bit. (Parent, public school)
**Acceptability**	
Acceptance of teachers conducting vision screening	Absolutely not. (Parent/guardian, private school) It needs to be done by a professional, zapatero a tus zapatos [stick to what you know]. (Parent/guardian, private school) A professional spends 5 years of their life getting educated. The health service provision at the school is very basic, as far as the school nurse goes. I don’t even allow my child to receive medication, unless it’s herbal tea, if there is the need. (Parent/guardian, private school) You would need a communication campaign to get parents on board, probably even the education minister on board. (NGO representative) Not at all reasonable. That would be the responsibility of the government to take care of. It is not for us to be in charge of. (Teacher, public school) It’s difficult because as a teacher, because in health, especially for vision, it’s difficult for us to give any advice on those matters. (Teacher, public school)
Stigma about wearing spectacles	Because not all the students are ready to use glasses. Some feel ugly if they use glasses. (Teacher, public school) At first I was scared because I thought I was going to be bullied for wearing glasses. (Student, public school)
Distrust of health services	There are a lot of requirements and legal processes to be done in order to begin any school health campaigns. (Parent, private school) It is important to explain the screenings to the parents so they can trust and be comfortable with it. (Parent, private school) There needs to be a proper explanation to the parents so they can understand what type of exam will be performed here at the school in order to allow it. So we know that the child is not going to be touched [inappropriately]. (Parent, private school)
**Accessibility**	
Economic accessibility	Of course cost is a barrier. If I am a worker, by law I have to contribute to the [health insurance] system and the waiting list for the subsidized system is long. (Parent, private school) Optical products are very expensive in Colombia. Glasses became a business so they are very expensive and even if the prescription is low the price is high. (Parent, private school) ‘My parents know it’s important to see well, but there is no money to buy glasses for me. (Female student, Public school) I was also prescribed with glasses, but there is no money in my house to afford them. (Male student, public school) Many people who live in strata one or two don’t even receive the minimum wage. As they don’t have a regular salary, their priorities would certainly not be eye health. (Eye-care practitioner)
Information accessibility	There’s one more thing about parents. Some of them think glasses damage or worsen eyesight. They don’t want their children using glasses, because their eyes will get worse. (NGO representative) Motivation from the parents and students, so they know how important eye health is. Giving information, statistics to the teacher that students cannot learn if they can’t read well enough. (Principal, private school) We have studied a lot what the main barriers [to eye care] are, and we think it is awareness of how important glasses are or why they are important. (NGO representative)
Geographic accessibility	All services are concentrated further north [of Bogotá]. Transport is difficult in Bogota because of the traffic, especially going to the north. (Parent, public school) When we ask parents why they don’t take the kids to the EPS service, they say it’s mainly because the places are very far or the appointments are very far [from their homes]. (NGO representative)
Quality	Schools have had vision screening where promises were not fulfilled which has led them [schools] to distrust any similar initiatives. These [the people who did the vision screening] are private optometrists or even salesmen who are not clinicians. (Eye-care practitioner) If you go private, you can’t trust the qualifications. (Teacher, public school)

AAAQ, availability, accessibility, acceptability and quality; NGO, nongovernmental organization; EPS, entidades promotoras de salud (health insurance organizations).

***Source*:** Prepared by the authors based on the analysis of the study data.

Key informants described an institutional culture in which staff members of the health and education ministries were hesitant to become involved in projects outside their jurisdictions, even though school health was a cross-sectoral area. Reported budgetary issues for visual acuity screening at both ministry and school-system levels had affected the availability of school eye care programs. Representatives from the Ministry of Education confirmed that collaboration between the two ministries at the policy level had been problematic, which affected the sustainability of school eye health screening interventions in the past. A representative from a nongovernmental organization described that lobbying health insurance organizations (namely entidades promotoras de salud) in Colombia is required to ensure the provision and follow-up of school-based eye care. Eight participants stated that eye health was not prioritized by the government. The Ministry of Health’s clinical practice guidelines for early detection of refractive error among children (22) recommends that teachers be trained to conduct vision screening in schools (p.42). However, an absence of policy structures that ensure public funds to deliver teacher training and school-based vision screening continue to prevent progress. Government representatives confirmed that school eye health interventions were largely focused on early childhood development, low vision, and inclusive education (through the Ministry of Education’s National Institute for the Blind or Instituto Nacional para Ciegos) rather than mass screening for refractive error.

#### Systems and resources.

Colombian schoolchildren have mandatory vision screening and proof of visual acuity at ages 4, 11, and 16 years, as a requirement for school entry. However, key informants and parents said that this was not enforced, particularly in public schools, which are unable to refuse children access to education. One government representative noted a strategy led by the health ministry called “I see well, I learn well” (Veo bien, aprendo bien). This strategy has three approaches to identifying children with vision problems: 1) nurses check visual acuity in schools; 2) optometry students check visual acuity in schools (in some regions only); or 3) teachers compile a list of children with possible vision problems. A government representative described the referral pathway for children identified with vision problems, yet highlighted that further regulation was still required. While the health insurance organizations follow up school referrals, the responsibility to bring children to these organizations for an eye examination lies with the parents. According to school staff, parents and children, eye care in schools, including visual acuity screening or eye health education, rarely occurred unless nongovernmental or private organizations were involved. A private school nurse said that she had not been trained to detect children with vision problems or conduct visual acuity screening. Resources to support school health in general in the public school system were even more limited. A public school parent described the importance of non-government assistance in the provision of school health, highlighting the inefficiency of the health system and poor regulation of school health.

### Acceptability

#### Acceptance of teachers conducting vision screening.

Discussing the possibility of training teachers to screen for visual acuity was divisive and revealed a clear concern of participants about adequate qualifications. Several respondents mentioned the phrase “zapatero a sus zapatos”, meaning “stick to what you know”, indicating a cultural belief that professionals should practise within their qualifications. When asked their thoughts about a teacher testing their children’s visual acuity, parents said that they would not be comfortable. Participants reported previous government initiatives that trained teachers in vision screening and identified the Health to Schools program as a cost-effective approach to detecting children with vision impairment. However, both programs had been discontinued.

Representatives from nongovernmental organizations stated that, while teacher training is theoretically an effective approach to enabling eye care, high-quality training, approval of the national optometrists’ association, and the health and education ministries is essential. Government representatives from the Ministry of Health considered the idea a cost-effective approach. Representatives from the education sector considered that training teachers in vision screening was outside the scope of their work. This perspective was echoed by teachers themselves.

#### Stigma related to spectacles.

Representatives of the government and nongovernmental organizations reported that spectacle use among children can result in stigmatization and bullying. However, stigma associated with wearing spectacles was not commonly reported among the children who participated in this study. Where stigma was discussed, it was attributed to cosmetic factors whereby spectacles are considered unattractive, and subsequently linked to fear of bullying.

#### Distrust of health services.

Negative perceptions of eye-care practitioners and health service providers were also identified. Distrust was linked to previous school eye-health interventions that had not delivered what they had promised and suspicion of commercial interests. Parents echoed this distrust, outlining that consent would have to be sought from them before conducting any eye health screenings or programs in school. Such views were linked to concerns for their child’s safety.

### Accessibility

#### Economic accessibility.

The average cost of spectacles was reported by key informants to be 80 000 Columbian pesos (COP) (US$ 21.23; 1 US$ = 3768.03 COP). A representative from the Ministry of Health stated that under the subsidized health insurance plan, up to COP 70 000 of the total spectacle cost is covered by the government, leaving either a small or no co-payment. We asked participants how much Colombian parents with lower incomes (strata one and two) would be willing to pay for their children’s spectacles, with key informants reporting a range of COP 10 000–40 000 and school staff reporting a range of COP 40 000–100 000. Representatives from the government and nongovernmental organizations considered subsidized spectacles were affordable. However, some middle-income parents (stratum four) with children at private school communicated that the cost of eye care was still a problem, citing barriers to accessing the subsidized system. Participants also recognized the difficulties that may be faced by parents and children in lower strata, or those working in the informal sector where basic economic needs are more pressing. A few public-school children in this study had been prescribed spectacles, but their parents had not been able to afford them.

#### Information accessibility.

Private-school children had much better eye-care knowledge than public-school children, particularly about the importance of wearing spectacles. School staff, parents, the eye-care practitioner, and representatives from the government and nongovernmental organizations all considered that parents, teachers, and the broader community require more access to information on the importance of child eye health and its link to education and development, as well as campaigns targeting misconceptions about spectacle use. Participants suggested that some parents were unlikely or less likely to perceive eye care as a priority without initiatives to raise awareness about eye health.

#### Geographic accessibility.

Although this study focused on the accessibility of school-based eye health programs, access to specialist eye care is an essential component of comprehensive eye care. Participants noted that in Bogotá, distance of treatment centers from peoples’ homes, lack of available transportation, and time away from work all contribute to people not accessing health or eye care. For individuals in lower strata, resources are limited, and vision does not rank high among other basic needs, such as food, education, or more pressing health issues.

### Quality

#### Qualification of eye-care practitioners.

Participants mentioned concerns about potentially unqualified eye-care practitioners from the private sector providing low-quality or inappropriate care to schoolchildren. This was linked to services that were seen as having a commercial interest.

## DISCUSSION

Our study provides evidence of factors that might facilitate or inhibit the appropriate adoption of eye health programs in Colombian schools. A rights-based framework (18) allowed us to reveal a range of complex systemic, economic and cultural barriers to children’s access to school-based eye health programs in Bogotá, extending beyond physical accessibility. Unlike low-income countries such as Nepal (23) and Sudan (24), where limited government budgets restrict the provision of government-funded school-based eye health programs, the problem in Colombia is related less to the availability of governmental funding, than to a lack of collaboration between the health and education ministries, as well as between health service providers and health insurance organizations. Previous studies point to the importance of cross-ministry and multisectoral support, working in conjunction with local authorities, hospitals, eye-care practitioners, nurses, teachers, and parents, and bolstered by a strong national policy, to ensure the success of school-based programs for eye care (25–27). Chile provides a good example of a school health program that includes eye health, which is run by the education ministry (9). As outlined, evidence of some cooperation between the Colombian ministries of health and education already exists in the requirement for children to undertake an eye check before starting a school year. However, issues on collaboration and accountability at the governance level continue to create barriers to improving child eye health and education outcomes. The Lancet Global Commission on Global Eye Health (9) noted that governing through strong leadership and clear policy, regulation, and accountability is crucial for improved eye health. Furthermore, the recently updated *Strategic Plan of the Pan American Health Organization 2020–2025* reaffirmed the CD53/11 Plan of Action for the Prevention of Blindness and Visual Impairment, which identifies health authority governance of visual health as a strategic line of action, including the development, updating and monitoring of national eye health policies and plans (10). In Colombia, increased collaboration, for example, a memorandum of understanding between the government ministries, may enhance responsibility and maintain accountability.

In a socioeconomically diverse context such as Colombia, where marked differences exist between public and private schools and health systems, it is important to consider discrimination and systemic inequality to have a holistic understanding of accessibility. Our study identified barriers related to the availability of health care professionals in both public and private schools as one of many factors that impede school-based eye care. Despite this, school eye health care may yet be preferable, as this approach seeks to overcome financial barriers that parents, especially those in lower socioeconomic strata, reported as a key factor preventing their children from accessing eye care. This finding supports others that suggest routine vision screening within schools can overcome poor uptake of care outside of the education system (11).

Using teachers as vision screeners is recognized as a cost-effective solution to identifying vision impairment in schoolchildren (11). It is favored in resource-restricted settings such as India (28), Peru (25), and Thailand (26). While teacher screening may theoretically be an appropriate solution in Colombia, our findings show that this approach was met with considerable reluctance among parents and teachers. Evidence from Chile shows that the legitimization of the role of teachers in the delivery of primary eye care can be attributed to the fact that the Chilean school eye health program is managed by the education ministry (29), rather than the health ministry as in Colombia. This suggests that policy-based barriers described in our study may need to be addressed before progress in school eye health can be made. Teachers could also be considered for alternative roles such as eye-health promotion or encouraging compliance with wearing spectacles. However, there is limited evidence on the effectiveness of such interventions (30). School nurses may provide a culturally acceptable pathway to school-based vision screening in Colombia. However, as shown in our case study, socioeconomic differences across the Colombian school system may be a barrier, as school nurses may be unavailable in public schools serving children from low strata.

Alternatively, in-school interventions could focus on: the provision or training of visiting nurses or health care professionals; health promotion initiatives aimed at reducing negative perceptions of health services and spectacles; and raising awareness of the effect of vision impairment on education and future employment (11). This approach may help correct the common perception among parents that eye health is not a priority. Acknowledging that trust needs to be established with parents, it is important to separate commercial interests and government-led health screenings.

Particular focus is required for people in the lowest socioeconomic stratum where the prevalence of presenting with visual impairment is highest (31). Considering the minimum hourly wage in Colombia is COP 10 200 per hour (32), most respondents said that spectacles were unaffordable and engagement with the health services was difficult due to work commitments and distance. Low-cost spectacles for children are needed when parents have to prioritize more immediate expenses over eye health. This is because Colombia’s subsidized health care plans do not always result in actual access to health care (33).

## Limitations

This study was conducted in the city of Bogotá and does not adequately represent issues faced elsewhere in the country. Purposive sampling may have led to selection bias; however, as this was a case study, our aim was not to obtain a representative sample, and thus the perspectives of the participants should be viewed with caution. Our study would have benefited from exploring the perspectives of representatives of nongovernmental organizations who work with children with vision-related disabilities. However, this was not possible at the time of data collection. While we made efforts to include wealthy and poorer people, obtaining the opinions of those from lower strata was challenging as participants were not available to participate. Similarly, no participants were of indigenous South American or African descent; therefore, the findings of our study may not represent these minority groups. Finally, the majority of our parent participants were women, most likley because mothers have the primary responsibility for children’s health, which may skew the data due to a lack of male respondents.

## Conclusion

Our study explored the many factors (barriers and facilitators) that affect school-based eye health programs in Bogotá, Colombia, including availability, accessibility, acceptability, and quality. We found poor cultural acceptance of training teachers as vision screeners. This key finding can help inform future policies and planning in the broader region. Our study also showed the need for a robust school eye health plan, and interventions that: improve cooperation between health and education ministries; distinguish between commercial interests and health service provision; address the lack of human resources while respecting professional qualifications; and raise awareness of the importance of eye health and its link to educational success.

## Disclaimer.

The authors are solely responsible for the views expressed in the manuscript, which may not necessarily reflect the opinion or policy of the *Revista Panamericana de Salud Pública / Pan American Journal of Public Health* and/or those of the Pan American Health Organization.

## References

[1] 1. Resnikoff S, Pascolini D, Mariotti SP, Pokharel GP. Global magnitude of visual impairment caused by uncorrected refractive errors in 2004. Bull World Health Organ. 2008;86(1):63–70. 10.2471/BLT.07.041210 PMC2647357 18235892

[2] 2. Hopkins S, Sampson GP, Hendicott PL, Wood JM. Vision problems and reduced reading outcomes in Queensland schoolchildren. Optom Vis Sci. 2017;94(3):345–52. 10.1097/OPX.0000000000001032 28079738

[3] 3. Ma X, Zhou Z, Yi H, Pang X, Shi Y, Chen Q, et al. Effect of providing free glasses on children’s educational outcomes in China: cluster randomized controlled trial. BMJ. 2014;349:g5740. 10.1136/bmj.g5740 PMC4172821 25249453

[4] 4. Salomao SR, Cinoto RW, Berezovsky A, Mendieta L, Nakanami CR, Lipener C, et al. Prevalence and causes of visual impairment in low–middle income school children in São Paulo, Brazil. Invest Ophthalmol Vis Sci. 2008;49(10):4308–13. 10.1167/iovs.08-2073 PMC6031127 18829856

[5] 5. Ministerio de Salud y Protección Social. Plan Decenal de Salud Pública PDSP, 2012–2021: La salud en Colombia la construyes tú [Ten-Year Public Health Plan PDSP 2012–2021: Health in Colombia is built by you]. Bogatá: Ministerio de Salud y Protección Social; 2012.

[6] 6. United Nations. International Covenant on Economic, Social and Cultural Rights. New York: UN; 1966. Available from: https://www.ohchr.org/Documents/ProfessionalInterest/cescr.pdf

[7] 7. United Nations. Convention on the Rights of the Child. New York; UN; 1989. Available from: https://www.ohchr.org/en/professionalinterest/pages/crc.aspx

[8] 8. United Nations Department of Economic and Social and Economic Affairs. Sustainable Development Goals 2015. New York: UN; 2015. Available from: https://sustainabledevelopment.un.org/?menu=1300

[9] 9. Burton MJ, Ramke J, Marques AP, Bourne RR, Congdon N, Jones I, et al. The Lancet Global Health Commission on Global Eye Health: vision beyond 2020. Lancet Glob Health. 2021;9(4):e489–e551. 10.1016/S2214-109X(20)30488-5 PMC7966694 33607016

[10] 10. Strategic Plan of the Pan American Health Organization 2020–2025: Equity at the heart of health. Washington, DC: Pan American Health Organization; 2020.

[11] 11. Burnett AM, Yashadhana A, Lee L, Serova N, Brain D, Naidoo K. Interventions to improve school-based eye-care services in low- and middle-income countries: a systematic review. Bull World Health Organ. 2018;96(10):682–94. 10.2471/BLT.18.212332 PMC6238998 30455516

[12] 12. Gilbert C, Minto H, Morjaria P, Khan I. Standard guidelines for comprehensive school eye health programs. Chippenham: Sightsavers International, London: London School of Hygiene and Tropical Medicine, Sydney: Brien Holden Vision Institute; 2016.

[13] 13. Education Policy & Data Centre. Economic inequality and schooling in Colombia 2013. Washington, DC: Education Policy & Data Centre; 2013. Available from: https://www.epdc.org/node/5836.htm

[14] 14. World Bank country and lending groups [Internet]. Washington, DC: The World Bank Group; 2021 Available from: https://datahelpdesk.worldbank.org/knowledgebase/articles/906519-world-bank-country-and-lending-groups

[15] 15. Muñoz JH, Salas JCD. Análisis econométrico espacial de las localidades de Bogotá y municipios del borde urbano [Spatial econometric analysis of Bogotá and urban municipalities]. Criterios. 2016;9(2):129–57.

[16] 16. Bogliacino F, Jiménez Lozano L, Reyes D. Socioeconomic stratification and stereotyping: lab-in-the-field evidence from Colombia. Int Rev Econ. 2018;65(1):77–118.

[17] 17. Ministerio de Salud y Protección Social. Comunicacion en las Regiones [Internet]. Bogatá: Ministerio de Salud y Protección Social; 2019. Available from: https://www.minsalud.gov.co/Regiones/Paginas/%E2%80%9CVeo-bien-aprendo-bien%E2%80%9D,-campa%C3%B1a-de-salud-visual-impulsada-por-minsalud.aspx

[18] 18. Committee on Economic Social & Cultural Rights. General Comment No.14, the right to the highest attainable standard of health. New York: United Nations; 2000.

[19] 19. Yin RK. Case study methods. In: Cooper H, Camic PM, Long DL, Panter AT, Rindskopf D, Sher KJ, editors. APA handbook of research methods in psychology, Vol. 2. Research designs: quantitative, qualitative, neuropsychological, and biological. Washington, DC: American Psychological Association; 2012.

[20] 20. Rubin HJ, Rubin IS. Qualitative interviewing: the art of hearing data. Thousands Oaks: Sage Publications Inc.; 2011.

[21] 21. Glaser BG, Strauss AL. The discovery of grounded theory: strategies for qualitative research. London: Routledge; 2009.

[22] 22. Guía de Práctica Clínica para la detección temprana, el diagnóstico, el tratamiento y el seguimiento de los defectos refractivos en menores de 18 años (Guía para Profesionales de la Salud. Guía No. 47) [Clinical practice guide for the early detection, diagnosis, treatment and follow-up of refractive defects in children under 18 years of age (Guide for health professionals. Guide no. 47)]. Bogata: Ministerio de Salud y Protección Social; 2016 Available from: https://www.minsalud.gov.co/sites/rid/Lists/BibliotecaDigital/RIDE/DE/CA/gpc-profesionales-defectos-refrectivos-menores-18anos.pdf

[23] 23. Adhikari S, Shrestha U. Validation of performance of certified medical assistants in preschool vision screening examination. Nepal J Ophthalmol. 2011;3(2):128–33. 10.3126/nepjoph.v3i2.5264 21876585

[24] 24. Alrasheed SH, Naidoo KS, Clarke-Farr PC. Childhood eye care services in South Darfur State of Sudan: learner and parent perspectives. Afr Vis Eye Health J. 2016;75(1). 10.4102/phcfm.v10i1.1767 PMC6244194 30456975

[25] 25. Latorre-Arteaga S, Gil-Gonzalez D, Enciso O, Phelan A, Garcia-Munoz A, Kohler J. Reducing visual deficits caused by refractive errors in school and preschool children: results of a pilot school program in the Andean region of Apurimac, Peru. Glob Health Action. 2014;7:22656. 10.3402/gha.v7.22656 PMC3925804 24560253

[26] 26. Teerawattananon K, Myint CY, Wongkittirux K, Teerawattananon Y, Chinkulkitnivat B, Orprayoon S, et al. Assessing the accuracy and feasibility of a refractive error screening program conducted by school teachers in pre-primary and primary schools in Thailand. PLoS ONE. 2014;9(6):e96684. 10.1371/journal.pone.0096684 PMC4057069 24926993

[27] 27. Alves Bezerra T, Tavares Aleixo A, de Freitas Macêdo Costa KN. Experience report from the partnership between health and education on the health at school program. J Nurs UFPE online. 2016;10(6):2262–6.

[28] 28. Priya A, Veena K, Thulasiraj R, Fredrick M, Venkatesh R, Sengupta S, et al. Vision screening by teachers in Southern Indian schools: testing a new “all class teacher” model. Ophthalmic Epidemiol. 2015;22(1):60–5. 10.3109/09286586.2014.988877 25495755

[29] 29. Barria Von-Bischhoffshausen F, Munoz B, Riquelme A, Ormeno MJ, Silva JC. Spectacle-wear compliance in school children in Concepcion Chile. Ophthalmic Epidemiol. 2014;21(6):362–9. 10.3109/09286586.2014.975823 25356984

[30] 30. Yi H, Zhang H, Ma X, Zhang L, Wang X, Jin L, et al. Impact of free glasses and a teacher incentive on children’s use of eyeglasses: a cluster-randomized controlled trial. Am J Ophthalmol. 2015;160(5):889–96.e1. 10.1016/j.ajo.2015.08.006 26275472

[31] 31. Luque LC, Naidoo K, Chan VF, Silva JC, Naduvilath TJ, Peña F, et al. Prevalence of refractive error, presbyopia, and spectacle coverage in Bogota, Colombia: a rapid assessment of refractive error. Optom Vis Sci. 2019;96(8):579–86. 10.1097/OPX.0000000000001409 31318796

[32] 32. Organisation for Economic Cooperation and Development. Real minimum wages: Colombia [Internet]. Paris: OECD; 2019. Available from: https://stats.oecd.org/Index.aspx?DataSetCode=RMW

[33] 33. García J. Closing the gap between formal and material health care coverage in Colombia. Health Hum Rights. 2016;18(2):49–65. PMC5394995 28559676

